# Antibacterial Activity of *Myristica fragrans* against Oral Pathogens

**DOI:** 10.1155/2012/825362

**Published:** 2012-08-28

**Authors:** Zaleha Shafiei, Nadia Najwa Shuhairi, Nordiyana Md Fazly Shah Yap, Carrie-Anne Harry Sibungkil, Jalifah Latip

**Affiliations:** ^1^Department of Clinical Oral Biology, Faculty of Dentistry, Universiti Kebangsaan Malaysia, Jalan Raja Muda Abdul Aziz, 50300 Kuala Lumpur, Malaysia; ^2^School of Chemical Sciences and Food Technology, Faculty of Science and Technology, Universiti Kebangsaan Malaysia, 43600 UKM Bangi, Selangor, Malaysia

## Abstract

*Myristica fragrans *Houtt is mostly cultivated for spices in Penang Island, Malaysia. The ethyl acetate and ethanol extracts of flesh, mace and seed of *Myristica fragrans* was evaluated the bactericidal potential against three Gram-positive cariogenic bacteria (*Streptococcus mutans *ATCC 25175, *Streptococcus mitis *ATCC 6249, and *Streptococcus salivarius *ATCC 13419) and three Gram-negative periodontopathic bacteria (*Aggregatibacter actinomycetemcomitans *ATCC 29522, *Porphyromonas gingivalis *ATCC 33277, and *Fusobacterium nucleatum *ATCC 25586). Antibacterial activities of the extracts was determined by twofold serial microdilution, with minimum inhibitory concentrations (MIC) ranging from 1.25 to 640 mg/mL and 0.075 to 40 mg/mL. The minimum bactericidal concentration (MBC) was obtained by subculturing method. Among all extracts tested, ethyl acetate extract of flesh has the highest significant inhibitory effects against Gram-positive and Gram-negative bacteria with mean MIC value ranging from 0.625 to 1.25 ± 0.00 (SD) mg/mL; *P* = 0.017) and highest bactericidal effects at mean MBC value ranging from 0.625 mg/mL to 20 ± 0.00 (SD) mg/mL. While for seed and mace of *Myristica fragrans*, their ethanol extracts exhibited good antibacterial activity against both groups of test pathogens compared to its ethyl acetate extracts. All of the extracts of *Myristica fragrans *did not show any antibacterial activities against *Fusobacterium nucleatum *ATCC 25586. Thus, our study showed the potential effect of ethyl acetate and ethanol extracts from flesh, seed and mace of *Myristica fragrans *to be new natural agent that can be incorporated in oral care products.

## 1. Introduction

Dental caries and periodontal disease are complex multifactorial diseases with dental plaque as their primary cause [1]. Gram-positive bacteria such as *Streptococcus mutans, Streptococcus sobrinus*, *Lactobacillus* species and some nonmutans streptococci are closely associated with caries formation [2–8]. Gram-negative bacteria such as *Aggregatibacter actinomycetemcomitans* is associated with aggressive periodontitis [9], while *Porphyromonas gingivalis, Tannerella forsythia, *and* Campylobacter rectus* are associated with chronic periodontitis in adult [10]. Caries and periodontal disease can be prevented by good maintenance of oral hygiene with the use of oral care products such as toothpaste, toothbrush, mouthwash, and oral paste that contain antimicrobial and anticariogenic properties. Nowadays, there are high interests in oral care products that are incorporated with medicinal plant extracts and are used extensively by the consumers due to low toxicity compared to oral care products containing antimicrobial agents such as triclosan, cetylpyridinium chloride, chlorhexidine, and amine fluorides that are reported to exhibit toxicity and cause staining of the teeth [11, 12]. It is well established that many metabolites produced by plant extracts such as tannins, terpenoids, alkaloids, and flavonoids provide new source of antimicrobial substances that help in combating new developing drug resistant pathogens [13–15].

*Myristica fragrans* Houtt (Family: Myristicaceae) locally known as buah pala in Malay is mostly cultivated for spices in Penang Island, Malaysia. It is composed of the skin, flesh, seed, and mace. Nutmeg is the seed kernel inside the fruit and mace is the fleshy red, net-like skin covering (aril) on the kernel [16]. The main constituents of *Myristica fragrans *have been found to be alkyl benzene derivatives (myristicin, elemicin, safrole, etc.), terpenes, alpha-pinene, beta-pinene, myristic acid, trimyristin [17–19], neolignan (myrislignan), and macelignan [20]. 

 The chemical constituents of *Myristica fragrans *have been investigated by scientists from various disciplines for hypolipidemic and hypocholesterolemic effects, antimicrobial, antidepressant, aphrodisiac, memory-enhancing, antioxidant, and hepatoprotective properties [16]. In traditional medicine, the seed kernel (nutmeg) is widely used as carminative, astringent, hypolipidaemic, antithrombotic, antiplatelet aggregation, antifungal, aphrodisiac [21], treating flatulence, nausea, and dyspepsia [22]. Mace is widely used as a flavouring agent, a hair dye, and a folk medicine. It also possesses antipapillomagenic, anticarcinogenic [23], and anti-inflammatory activities [24].

 In dentistry application, macelignan, an active compound from seed had strong anticariogenic activity, possessed antibacterial effect against oral microorganisms such as *Streptococcus *species, and *Lactobacillus *species, and exhibited weak activity for *Actinomyces viscosus*, *Porphyromonas gingivalis* and *Staphylococcus aureus* [20]. Narasimhan and Dhake [25] reported that trimyristin, an active compound obtained from seed of *Myristica fragrans,* also exhibited good antibacterial properties against Gram-positive and Gram-negative bacteria. 

So far, there is a lack of scientific reports that indicate the antimicrobial activities of this plant, especially for crude extracts of each parts of *Myristica fragrans *against oral pathogens. Hence, this study was aimed at investigating the antimicrobial activities of ethyl acetate and ethanol extracts of flesh, seed, and mace of *Myristica fragrans *against both Gram-positive and Gram-negative oral pathogenic bacteria. Since there is no study has been published to indicate the potential antibacterial activities of the crude extracts, thus, the reference strains were used rather than clinical isolates to investigate the antibacterial activities of the extracts tested. 

## 2. Materials and Methods 

### 2.1. Plant Materials

The freshly *Myristica fragrans* Houtt fruits were purchased from the local market at Balik Pulau, Penang Island, Malaysia. The *Myristica fragrans* were cut and divided into flesh, mace, and seed. Then, each part was dried for 7 days at room temperature (25°C). The dried samples were weighed, milled, ground into small pieces, and kept away from heat, moisture, and sunlight. The plant was authenticated at the *Forest Research *Institute Malaysia (FRIM) Kepong, Malaysia with voucher specimen number PID 210712-15 and deposited in FRIM Kepong Herbarium.

### 2.2. Preparation of Crude Extract

The dried samples of each part of *Myristica fragrans *(flesh, mace, and seed) were extracted using Soxhlet extraction (Thermo-Fisher, UK) method with ethyl acetate (semipolar) (Merck, USA) and ethanol (polar) (Merck, USA) solvents, successively. The extraction processes were carried out for 3 days for each of the samples to obtain crude extracts which were concentrated under reduced pressure by using a rotary evaporator (Eyela N-1000, Japan). The resulting pellet was finally pounded to dryness at room temperature to produce a powdery crude ethyl acetate and ethanol extracts, respectively. The crude extracts were then stored at −20°C for use in further studies.

### 2.3. Preparation of Bacterial Suspension

We used three facultative anaerobic Gram-positive cariogenic bacteria, which included *Streptococcus mutans* ATCC 25175, *Streptococcus mitis* ATCC 6249, and *Streptococcus salivarius* ATCC 13419 and three obligate anaerobic Gram-negative periodontopathic bacteria, *Aggregatibacter actinomycetemcomitans *ATCC 29522, *Porphyromonas gingivalis *ATCC 33277, and *Fusobacterium nucleatum *ATCC 25586 (ATCC, USA) in the present study. The bacterial stock cultures (stored at −80°C freezer) were obtained from Department of Clinical OralBiology, Microbiology Laboratory, Facultyof Dentistry, Universiti Kebangsaan Malaysia (UKM), Malaysia. The bacterial stock cultures were thawed and placed in brain heart infusion (BHI) (Oxoid, England) broth for Gram-positive bacteria and supplement-brain heart infusion (S-BHI) (Oxoid, England) which was supplemented with yeast extract (Scharlau, Spain), hemin (Merck, Germany), and vitamin K (Merck, Germany) broth for Gram-negative bacteria. All the broth with bacterial growth were incubated at 37°C, 5% CO_2_ (Shel Lab, USA) for 24 hours (facultative anaerobic) or 48 hours (obligate anaerobes). 

The bacteria were then standardized for their CFU/mL using respective filler broths (BHI broth for Gram-positive bacteria and S-BHI for Gram-negative bacteria) by using a spectrophotometer (Metertech SP-830, Taiwan). The turbidity of bacterial suspension was adjusted to absorbance (A) reading within the range of 0.08 to 0.10 at OD 625 nm which was equivalent to 1-2 × 10^8^ CFU/mL [26, 27]. 

The screening test using agar well-diffusion test of the extracts (50 *μ*L; at concentrations of 20 mg/mL and 5 mg/mL) failed to get zone of inhibition against tested bacteria due to poor diffusion of the extracts throughout the agar. Therefore, the direct contact using broth microdilution was performed.

### 2.4. Determination of MIC and MBC

The minimum inhibitory concentrations (MICs) of ethyl acetate and ethanol extracts of each part of *Myristica fragrans* against tested oral pathogens were determined using a broth microdilution method on 96-well microtiter plates (Sigma-Aldrich, Malaysia) as described by Basri et al. [28] with some modifications. Two stock solutions of the extracts at a concentration of 160 mg/mL and 2560 mg/mL were prepared with sterile distilled water due to different susceptibility of oral bacteria tested against the extracts. All the extract solutions were mixed till homogenous and sterilized by filtering through 0.45 *μ*m membrane filter paper (Jet Biofil, Canada). 

 Approximately 50 *μ*L of the prepared extract was added to the first well containing 50 *μ*L of sterile BHI broth (for Gram-positive bacteria) or S-BHI broth (for Gram-negative bacteria) and serially diluted by twofold method, resulting in final concentrations ranging from 0.075 g/mL to 40 mg/mL and 1.25 mg/mL to 640 mg/mL, respectively (from the 2nd well to the 10th well). From the 10th well, 50 *μ*L of solution was pipetted out and disposed off. Subsequently, 50 *μ*L of bacterial suspension containing 1-2 × 10^8^ CFU/mL was added into the 1st to 11th well. The 11th well served as positive control, containing 50 *μ*L BHI (for Gram-positive bacteria) or S-BHI broth (for Gram-negative bacteria) and 50 *μ*L of bacterial suspension. The 12th well contained 50 *μ*L broth (BHI or S-BHI) and 50 *μ*L of tested extractwith no bacterial inoculation and served as negative control. Different concentrations of tetracycline (for Gram-positive) and metronidazole (for Gram-negative bacteria) ranging from 0.01 to 5 *μ*g/mL used as positive control were also prepared by serial dilution in (BHI or S-BHI) agar, respectively. Fresh BHI or S-BHI broth used as negative control. 

The microtiter plate was then incubated at 37°C, 5% CO_2_ for 24 hours (for Gram-positive bacteria) or 48 hours (for Gram-negative bacteria) in an anaerobic jar. The procedures were then repeated thrice (technical replicates) for each extract, and the whole experiment was duplicated to ensure uniformity of results (biological replicates). A dye indicator was added to determine the bacterial growth by using 20 *μ*L 3-(4,5-dimethyl-2-thiazolyl) 2,5-diphenyl-2H-tetrazolium bromide (MTT, 1 mg/mL) (Merck, Germany) for Gram-positive and 40 *μ*L 2,3,5-triphenyltetrazolium chloride (TTC, 2 mg/mL) (Merck, Germany) for Gram-negative bacteria. The plate was incubated for another 2 hours in the dark. The lowest concentration of extract with (no visible growth) after colour changes were considered as the MIC value [26–29]. 

The minimum bactericidal concentration (MBC) value was determined by subculturing on BHI or S-BHI agar plates of each well component that was not showing visible indicator changes. The least concentration which showed no visible growth on the agar plate after incubation period was considered as the MBC value [26–29]. 

If the MIC and MBC values cannot be obtained as the bacteria was still able to growth after incubation (in broth microdilution and on agar plates), the results were presented as ND (not detectable). 

### 2.5. Statistical Analysis

All the data were expressed as the mean value ± standard deviation (SD). Data were analysed using IBM SPSS Statistics 20 (New York, USA, 2011) for nonparametric analysis of variance. Kruskal-Wallis and Mann-Whitney tests were used to analyze the significance difference of MIC and MBC of the extracts towards tested oral bacteria. Difference was considered statistically significant at *P* < 0.05. 

## 3. Results 

### 3.1. Percentage Yield of Crude Extract 

About 492.71 g dried flesh, 137.45 g dried mace and 419.74 g dried seed of *Myristica fragrans *were extracted with ethyl acetate and ethanol solvents using Soxhlet extractor to obtain semipolar and polar crude extracts, respectively. Semipolar and polar crude extracts were identified to be highest in seed (9.85%; 11.16%), followed by mace (8.90%; 9.6%) and flesh (5.05%; 4.08%), respectively (Table 1).

### 3.2. Determination of MIC and MBC Values

The minimum inhibitory concentrations (MICs) and minimum bactericidal concentrations (MBCs) of ethyl acetate and ethanol extracts from flesh, mace, and seed of *Myristica fragrans* against oral pathogens were specified in Tables 2 and 3. The low MIC and MBC values indicated that the extract has strong antibacterial activity. The result revealed that ethyl acetate and ethanol extracts of *Myristica fragrans* flesh possess good antibacterial activities against both Gram-positive and Gram-negative bacteria tested compared to the crude extracts of seed and mace (Table 2). With the exception of *Fusobacterium nucleatum* ATCC 25586, the minimum inhibitory concentrations assay indicated that the two groups of bacteria were susceptible to the extracts at very high concentrations with ethyl acetate extract of flesh has highest inhibitory effect against tested bacteria with MIC values ranging from 0.625 to 1.25 ± 0.00 (SD) mg/mL, followed by ethanol extract of flesh (mean MIC values ranging from 5 to 10 ± 0.00 (SD) mg/mL) and the other extracts tested. Ethanol extract of seed possess good antibacterial activities against the test bacteria compared to its ethyl acetate extract. While antibacterial activities of ethanol extract of mace was slightly lower compared to the other extracts with *Streptococcus mitis* ATCC 6249 having the highest minimum inhibitory effect at a mean concentration of 5 ± 0.00 (SD) mg/mL. The extract was less potent than the standard antibiotic, tetracycline or metronidazole, to which the bacteria were highly susceptible at 5 *μ*g/mL. 

 Of all the microorganisms, *Fusobacterium nucleatum* ATCC 25586 was found to be resistant to all the extracts tested at the highest concentration (160 mg/mL). The result was displayed as ND (not detectable). 

Minimum bactericidal concentrations (MBCs) of the extracts tested were expectedly higher than the minimum inhibitory concentrations (MICs). The MIC/MBC ratio indicated that the extract is bacteriostatic at lower concentration, while at higher concentration, it is bactericidal. While MIC equals MBC, the similarity revealed that the extract possesses potent antibacterial components against the test bacteria as showed by ethyl acetate extract of flesh against *Streptococcus salivarius* ATCC 13419 with mean MIC and MBC value of 0.625 ± 0.00 (SD) mg/mL (Table 3). The antibacterial activity of the ethyl acetate and ethanol extracts from flesh, seed, and mace of *Myristica fragrans* indicated a broad-spectrum and great therapeutic potential of the plant as anticariogenic and antiperiodontopathic agents.

## 4. Discussion

The antibacterial activities of ethyl acetate and ethanol extracts of flesh, seed, and mace *Myristica fragrans* Houtt were investigated in the present study. The crude extracts were prepared using polarity of the solvent by Soxhlet extractor as suggested by Prabu et al. [30]. Usually water, methanol, ethanol, ethyl acetate, hexane, or an aqueous ethanol-based solvent were used to prepare plant or plant-derived extracts. Ethanol was selected as polar solvent instead of methanol due to low toxicity and ethyl acetate also have low toxicity was chosen as a semipolar solvent. 

Due to poor diffusion of the extract into the surrounding agar from the wells, the screening test using agar well-diffusion assay failed to show the inhibition zones against tested bacteria. Broth microdilution method by using indicator dyes provides a more accurate determination of the MIC and the antibacterial activity of the extract, than the disc or agar well diffusion methods. Under liquid conditions, the microbial cells are in direct contact with extract, and the MIC values indicated the definite nature of the antibacterial activities of the extracts [31]. 

All of the ethyl acetate and ethanol extracts of flesh, seed, and mace of *Myristica fragrans* did not show any antibacterial activities against *Fusobacterium nucleatum* ATCC 25586, indicating that the crude extract might be insufficient extract concentration to give antibacterial effect or *Fusobacterium nucleatum *itself was resistant towards both ethyl acetate and ethanol extracts of *Myristica fragrans. *So far, no study of any extract of *Myristica fragrans *was done against *Fusobacterium nucleatum* ATCC 25586 for comparison. However, crude extracts (methanol and aqueous) from seed of *Garcinia kola* [32] and Brazilian propolis [33] have capability to kill *Fusobacterium nucleatum.*

With the exception of *Fusobacterium nucleatum* ATCC 25586, the results showed that the crude extracts of flesh, seed, and mace of *Myristica fragrans* had significant antibacterial potency against both Gram-positive cariogenic and Gram-negative periodontopathic bacteria tested. We found that among all extracts tested, ethyl acetate extract of flesh has the highest significant inhibitory effects (mean MIC value ranging from 0.625 to 1.25 ± 0.00 (SD) mg/mL; *P* = 0.017) and highest bactericidal effects at mean MBC values ranging from 0.625 mg/mL to 20 ± 0.00 (SD) mg/mL compared to the other extracts against bacteria tested. The bactericidal effects were extremely higher against Gram-positive cariogenic bacteria than Gram-negative periodontopathic bacteria, with *Streptococcus salivarius* ATCC 13419 is the most susceptible with the mean MIC and MBC of 0.625 ± 0.00 (SD) mg/mL. While for seed and mace of *Myristica fragrans*, their ethanol extracts exhibited good antibacterial activity against both groups of test pathogens compared to its ethyl acetate extracts but not as effective as the standard antibiotic used for comparison. Narasimhan and Dhake [25] extracted the powdered seed of *Myristica fragrans* with chloroform to obtain trimyristin, which on saponification yielded myristic acid and petroleum ether to obtain myristicin. All the constituents isolated from the seed exhibited good antibacterial activity against selected Gram-positive and Gram-negative bacteria. Perhaps, the antibacterial activities observed from ethyl acetate extract of seed in our studies were exhibited by trimyristin and myristicin.

However, findings from the present study showed that crude extract (either ethyl acetate or/and ethanol extracts) of all parts of *Myristica fragrans *exhibited lower antibacterial activity compared to the bioactive compound. The obtained result is similar to the finding of Chung et al. [20] showing that macelignan, an active compound from seed of *Myristica fragrans* exhibits strong antibacterial activity against *Streptococcus mutans *with MIC and MBC value of 3.9 *μ*g/mL, and 7.8 *μ*g/mL, respectively. Malabaricone C isolated from *Myristica fragrans *(seed) irreversibly inhibits Arg-gingipain by 50% at a concentration of 0.7 *μ*g/mL and selectively suppressed *Porphyromonas gingivalis *growth [34]. However, some previous studies reported that the crude extract of some medicinal plant showed higher inhibitory effects as active compound. Didry and coworkers [35] revealed that chloroform extract of the aerial parts of *Drosera peltate* exhibited greatest activity against *Streptococcus mutans* and *Streptococcus sobrinus* (MIC of 31.25 and 15.625 *μ*g/mL, resp.). While methanol extract of *Hamamelis virginiana *had the greatest activity against *Porphyromonas* sp., *Prevotella *sp., and *Actinomyces odontolyticus* with MIC value less than 512 *μ*g/mL [36]. 

Although the antibacterial activities of the crude extracts of each part of *Myristica fragrans* were considerably low, the significant effect of antibacterial activities of each part of *Myristica fragran*s indicated its medicinal potential that could be used against oral pathogens. Its ethyl acetate and ethanol extracts at different concentration, possess inhibitory effect indicating a broad spectrum of antimicrobial activity. Thus, further studies should be conducted to evaluate the potential antibacterial effect of ethyl acetate and ethanol extracts of each parts of *Myristica fragrans *especially flesh. To date, there was no study published about antibacterial potential of flesh of *Myristica fragrans* for comparison. Therefore, our study is a pioneer study about antibacterial activity of *Myristica fragrans* flesh against oral pathogens. 

## 5. Conclusion

Based on the current findings, it was proved that both ethyl acetate and ethanol crude extracts from flesh, seed, and mace of *Myristica fragrans* exhibited good potential against oral pathogens. Ethyl acetate extract of flesh has strong antibacterial activity compared to the other extracts. While, ethanol extracts of seed and mace of *Myristica fragrans* gave higher inhibitory and bactericidal activities than their ethyl acetate extracts. The antibacterial activities of the extracts against both Gram-positive cariogenic and Gram-negative periodontopathic bacteria have confirmed its broad-spectrum antibacterial activity. Thus, *Myristica fragrans *should be considered having beneficially potential in dentistry field as oral care products such as toothpaste and mouthwash. In addition, further studies are needed to be carried out to isolate and identify the active compounds of each part of *Myristica fragrans* and their influence in disruption of planktonic or biofilm formation for prevention and control of caries and periodontal diseases.

## Figures and Tables

**Table 1:** Total yield of ethyl acetate and ethanol extracts of *Myristica fragrans*.

Sample	Extraction solvent	Dried weight (g)	Total yield (g)	Percentage of total yield (%)
Flesh	Ethyl acetate	492.71	29.81	6.05
	Ethanol		20.13	4.08
Seed	Ethyl acetate	419.74	41.34	9.85
	Ethanol		46.86	11.16
Mace	Ethyl acetate	137.45	12.23	8.90
	Ethanol		13.25	9.60

**Table 2:** Mean MIC values of ethyl acetate and ethanol extracts from flesh, seed, and mace of *Myristica fragrans* against oral pathogens.

		Mean minimum inhibitory concentration		
Bacteria species	Extracts	(MIC) value ± 0.00 (SD) (mg/mL)		
		Flesh	Seed	Mace
*Streptococcus mutans* ATCC 25175	Ethyl acetate	1.25	20	ND
	Ethanol	10	10	20
*Streptococcus mitis* ATCC 6249	Ethyl acetate	0.625	20	20
	Ethanol	5	5	5
*Streptococcus salivarius* ATCC 13419	Ethyl acetate	0.625	80	ND
	Ethanol	5	20	40
*Aggregatibacter actinomycetemcomitans* ATCC 29522	Ethyl acetate	1.25	20	20
	Ethanol	10	20	40
*Porphyromonas gingivalis* ATCC 33277	Ethyl acetate	1.25	160	ND
	Ethanol	5	10	20
*Fusobacterium nucleatum* ATCC 25586	Ethyl acetate	ND	ND	ND
	Ethanol	ND	ND	ND

Lowest MIC value indicates the highest inhibitory effect.

ND: no detectable data.

**Table 3:** Mean MBC values of ethyl acetate and ethanol extracts of seed, flesh, and mace of *Myristica fragrans *against oral pathogens.

		Mean minimum bactericidal concentration		
Bacteria species	Extracts	(MBC) value ± 0.00 (SD) (mg/mL)		
		Flesh	Seed	Mace
*Streptococcus mutans* ATCC 25175	Ethyl acetate	2.5	40	ND
	Ethanol	10	20	40
*Streptococcus mitis* ATCC 6249	Ethyl acetate	1.25	40	40
	Ethanol	10	20	10
*Streptococcus salivarius* ATCC 13419	Ethyl acetate	0.625	320	ND
	Ethanol	10	80	640
*Aggregatibacter actinomycetemcomitans* ATCC 29522	Ethyl acetate	20	40	ND
	Ethanol	20	40	80
*Porphyromonas gingivalis* ATCC 33277	Ethyl acetate	10	320	ND
	Ethanol	40	40	40
*Fusobacterium nucleatum* ATCC 25586	Ethyl acetate	ND	ND	ND
	Ethanol	ND	ND	ND

Lowest MIC value indicates the highest inhibitory effect.

ND: no detectable data.
